# Low-molecular-weight heparin venous thromboprophylaxis in critically ill patients with renal dysfunction: A subgroup analysis of the PROTECT trial

**DOI:** 10.1371/journal.pone.0198285

**Published:** 2018-06-01

**Authors:** Menaka Pai, Neill K. J. Adhikari, Marlies Ostermann, Diane Heels-Ansdell, James D. Douketis, Yoanna Skrobik, Ismael Qushmaq, Maureen Meade, Gordon Guyatt, William Geerts, Michael W. Walsh, Mark A. Crowther, Jan O. Friedrich, Lisa Burry, Rinaldo Bellomo, Nilton Brandão da Silva, Rubens Costa Filho, Michael J. Cox, Suzana Alves Silva, Deborah J. Cook, on behalf of the PROTECT (Prophylaxis for Thromboembolism in Critical Care Trial) Investigators

**Affiliations:** 1 Department of Medicine, McMaster University, Hamilton, Canada; 2 Hamilton Regional Laboratory Medicine Program, Hamilton, Canada; 3 Department of Pathology and Molecular Medicine, McMaster University, Hamilton, Canada; 4 Department of Critical Care Medicine, Sunnybrook Health Sciences Centre, Toronto, Canada; 5 Interdepartmental Division of Critical Care Medicine, University of Toronto, Toronto, Canada; 6 Department of Critical Care, Guys and St Thomas Hospital, London, England; 7 Department of Health Research Methods, Evidence, and Impact, McMaster University, Hamilton, Canada; 8 Department of Medicine, Hôpital Maisonneuve Rosemont, Montréal, Canada; 9 Department of Medicine, King Faisal Specialist Hospital & Research Center, Jeddah, Saudi Arabia; 10 Department of Medicine, University of Toronto, Toronto, Canada; 11 Mount Sinai Hospital, Toronto, Canada; 12 Australian and New Zealand Intensive Care Research Centre (ANZIC-RC), Monash University, Melbourne, Australia; 13 Moinhos de Vento Hospital, Porto Alegre, Brazil; 14 Pró Cardíaco Hospital, PROCEP, Rio de Janeiro, Brazil; 15 St. John's Mercy Medical Center, St. Louis, Missouri, United States of America; Maastricht University Medical Center, NETHERLANDS

## Abstract

**Introduction:**

There is concern about excessive bleeding when low-molecular-weight heparins (LMWHs) are used for venous thromboembolism (VTE) prophylaxis in renal dysfunction. Our objective was to evaluate whether LMWH VTE prophylaxis was safe and effective in critically ill patients with renal dysfunction by conducting a subgroup analysis of PROTECT, a randomized blinded trial.

**Methods:**

We studied intensive care unit (ICU) patients with pre-ICU dialysis-dependent end-stage renal disease (ESRD; pre-specified subgroup; n = 118), or severe renal dysfunction at ICU admission (defined as ESRD or non-dialysis dependent with creatinine clearance [CrCl] <30 ml/min; *post hoc* subgroup; n = 590). We compared dalteparin, 5000 IU daily, with unfractionated heparin (UFH), 5000 IU twice daily, and considered outcomes of proximal leg deep vein thrombosis (DVT); pulmonary embolism (PE); any VTE; and major bleeding. Adjusted hazard ratios [HR] were calculated using Cox regression.

**Results:**

In patients with ESRD, there was no significant difference in DVT (8.3% vs. 5.2%, p = 0.76), any VTE (10.0% vs. 6.9%; p = 0.39) or major bleeding (5.0% vs. 8.6%; p = 0.32) between UFH and dalteparin. In patients with severe renal dysfunction, there was no significant difference in any VTE (10.0% vs. 6.4%; p = 0.07) or major bleeding (8.9% vs. 11.0%; p = 0.66) but an increase in DVT with dalteparin (7.6% vs. 3.7%; p = 0.04). Interaction p-values for comparisons of HRs (ESRD versus not) were non-significant.

**Conclusions:**

In critically ill patients with ESRD, or severe renal dysfunction, there was no significant difference in any VTE or major bleeding between UFH and dalteparin. Patients with severe renal dysfunction who received dalteparin had more proximal DVTs than those on UFH; this finding did not hold in patients with ESRD alone.

## Introduction

Patients admitted to an intensive care unit (ICU) are at high risk for developing venous thromboembolism (VTE), which comprises deep vein thrombosis (DVT) and pulmonary embolism (PE). Despite prophylaxis with unfractionated heparin (UFH), 5% of patients develop proximal DVT, and this rate is higher among patients with septic shock [1, 2]. VTE prophylaxis with low-molecular-weight heparin (LMWH) is an appealing option in ICU patients due to administration ease (daily dosing), availability of pre-filled syringes (reducing the chance of medication error), and a lower incidence of heparin-induced thrombocytopenia than UFH [3, 4]. However, LMWH use in ICU patients may be concerning because, unlike UFH, these agents are cleared mainly by the kidney [5], and a high proportion of ICU patients will have acute or chronic renal dysfunction. Such patients also exhibit complex imbalances between procoagulant and anticoagulant systems related to nephrotic loss of natural anticoagulants, impaired fibrinolysis, administration of exogenous anticoagulants (i.e., during dialysis), and uremic platelet dysfunction [6].

Clinically important bioaccumulation occurs in patients with renal dysfunction who receive therapeutic doses of LMWHs (e.g., dalteparin 12000–18000 IU daily), which are typically used to treat acute VTE. These doses are avoided in patients with *severe renal dysfunction*, which we define as either creatinine clearance (CrCl) <30 mL/min without dialysis dependence, or dialysis-dependent end-stage renal disease (ESRD) [7, 8]. On the other hand, in such patients who receive prophylactic-dose LMWH (e.g., dalteparin 5000 IU daily), there is evidence from small observational studies of no bioaccumulation when such low-dose regimens are used for 7–10 days [9–13].

Few studies have evaluated LMWHs for VTE prophylaxis in ICU patients with severe renal dysfunction. In a pilot study of 19 ICU patients with renal impairment, including several patients needing acute hemodialysis, bioaccumulation was not observed with prophylactic LMWH (dalteparin, 5000 IU daily) [11]. Similarly, in a multicentre prospective cohort study of 138 ICU patients with an estimated CrCl <30 mL/min, no bioaccumulation occurred with prophylactic dalteparin [10]. Major bleeding occurred in 7% of these patients, but all had low trough anti-factor Xa levels (≤0.18 IU/mL). Moreover, none of these studies assessed bioaccumulation or clinical outcomes when LMWH was compared to VTE prophylaxis with UFH, which is dependent on non-renal mechanisms for clearance.

To further evaluate the premise that prophylactic LMWH should be avoided in patients with severe renal dysfunction, we conducted a subgroup analysis of PROTECT, a randomized trial comparing dalteparin (5000 IU daily) with UFH (5000 IU twice-daily) for VTE prophylaxis in critically ill patients [2]. Our primary pre-specified objective was to compare the efficacy (VTE) and safety (major bleeding) of dalteparin and UFH in patients with dialysis-dependent ESRD before ICU admission. Our secondary *post hoc* objective was to conduct the same comparisons in patients with a broader spectrum of renal impairment, encompassing those with dialysis-dependent ESRD or CrCl <30 mL/min without dialysis.

## Materials and methods

### Study design

The PROTECT study was a multicentre, randomized, blinded controlled trial comparing the LMWH dalteparin, 5000 IU daily, to UFH, 5000 IU twice-daily, for the prevention of VTE in critically ill ICU patients.[2] The primary outcome in PROTECT was proximal leg DVT; secondary outcomes included major bleeding, PE, and any VTE. The complete date range for participant recruitment and follow-up was May 2006 to June 2010. PROTECT was approved by research ethics boards of all participating hospitals (S1 Appendix).

We conducted a primary pre-specified subgroup analysis in patients with ESRD, defined by pre-ICU (baseline) dialysis dependence, in whom we postulated *a priori* based on our previous work [10] that LMWH would not be associated with higher rates of VTE or major bleeding than UFH. A secondary *post hoc* analysis was done in patients with *severe renal dysfunction* at ICU admission, defined as dialysis-dependent ESRD or non-dialysis-dependent but with an estimated CrCl <30 mL/min. We considered but rejected a secondary post hoc analysis in patients not on dialysis but with estimated CrCl <30 mL/min, reasoning that the larger group of patients with severe renal dysfunction was more relevant to clinicians. For these subgroup analyses, there was no sample size calculation at the time the main trial was designed.

### Patients

Patients with the following inclusion criteria were enrolled: adults (age ≥18 years); body weight >45 kg; and expected ICU length of stay >72 hours. Exclusion criteria were major trauma; neurosurgery; orthopedic surgery; need for therapeutic anticoagulation; unfractionated heparin administration in the ICU for 3 days; contraindication to heparin or blood products; pregnancy; life-support limitation; or enrollment in a related trial. Research coordinators obtained written informed consent from all patients or their surrogates, as approved by research ethics boards of all participating hospitals. Patients were followed until the time of death in the hospital or discharge.

ESRD was defined as dependence on hemodialysis or peritoneal dialysis prior to ICU admission. Patients with severe renal dysfunction had either ESRD or CrCl <30 mL/min without dialysis dependence at the time of ICU admission, estimated by the Cockroft-Gault equation [14]. Patients with an initial CrCl <30 mL/min whose renal function improved during the ICU stay were retained within the severe renal dysfunction group, whereas patients with a CrCl ≥30 mL/min at the time of ICU admission who developed worsening renal function during the ICU stay were not included in the severe renal dysfunction group.

The subgroup analysis was designed by the authors and received approval from the PROTECT study Steering Committee. Funding for PROTECT was provided by the Canadian Institutes of Health Research, the Australian and New Zealand College of Anaesthetists Research Foundation, and the Heart and Stroke Foundation of Canada. Study drugs were provided by Pfizer and Eisai. Neither the funders nor the drug manufacturers played any role in the design or conduct of the trial or in the analysis or interpretation of the data. This trial is registered at ClinicalTrials.gov (identifier: NCT00182143).

### Anticoagulant regimen

Using a centralized electronic system, local research pharmacists randomly assigned enrolled patients to receive either subcutaneous LMWH (dalteparin 5000 IU daily) or UFH (5000 IU twice daily). Randomization was stratified according to centre and type of admission (medical versus surgical) with the use of undisclosed variable block sizes in a 1:1 ratio. Research pharmacists prepared identical syringes for subcutaneous injection of either dalteparin daily plus placebo daily (for parallel group twice-daily injections), or of UFH twice daily for the duration of the ICU stay. Patients, family members, clinicians, research personnel, ultrasonographers, and outcome adjudicators were all unaware of study-group assignments.

### Outcomes

For this study, the outcomes were proximal DVT, PE, any VTE, and major bleeding. Proximal DVT was defined as a new non-compressible vein segment of the popliteal or more proximal veins of the leg. All patients had routine screening bilateral leg venous ultrasound within 48 hours of study enrolment and twice weekly thereafter, as well as when clinically indicated. DVT diagnosed on the first screening ultrasound was considered prevalent DVT and was not included as a study outcome, whereas DVT detected subsequently was considered incident DVT and included as an outcome. Catheter-related DVT was classified as DVT according to its location (arm or leg). Routine screening for PE was not done. PE was defined as definite (characteristic intraluminal filling defect on chest computed tomography, a high probability ventilation–perfusion scan or autopsy finding), probable (high clinical suspicion and either no test results or non-diagnostic results on non-invasive testing), possible (clinical suspicion and a non-diagnostic test), or absent (negative tests). For this study, PE included definite, probable, or possible PE.

Major bleeding was defined as hemorrhage at a critical site (e.g., intracranial), necessitating a major therapeutic intervention (e.g., surgery), causing hemodynamic compromise, requiring 2 units of red-cell concentrates, or resulting in death. Minor bleeding was defined as bleeding that did not fulfill the criteria for major bleeding (e.g., heparin injection-site hematoma).

All VTE and major bleeding outcomes were independently adjudicated [2, 15, 16]. In formal calibration exercises, there was high concordance with respect to all outcomes [15, 16]. Thereafter, we randomly assigned each outcome to 2 adjudicators (or 4 adjudicators in the case of PE) who were unaware of randomized assignment and of one another’s assessments. Consensus was obtained for all outcomes with continued high levels of agreement throughout the trial.

### Statistical analysis

Descriptive statistics (mean, standard deviation [SD]; median, inter-quartile range [IQR]; number, percentage) were used to summarize baseline characteristics. The incidences of VTE (proximal DVT, PE, any VTE) and major bleeding according to treatment allocation (dalteparin or UFH) were reported in patients with ESRD vs. no ESRD and, in a different analysis, in patients with severe renal dysfunction (ESRD or CrCl <30 mL/min without dialysis dependence) vs. no severe renal dysfunction (CrCl ≥30 mL/min).

Hazard ratios (HRs) and corresponding 95% confidence intervals (CIs) and p-values for the effect of dalteparin versus UFH on VTE and major bleeding outcomes in each of these subgroups were obtained from Cox regression analysis, stratified by centre and medical/surgical admission. For these analyses, all patients were included in a Cox regression model that included the effect of baseline ESRD (or ESRD or CrCl <30 mL/min without dialysis dependence in a second model) on VTE and bleeding outcomes plus its interaction with study drug (LMWH or UFH). Hazard ratios for VTE events were adjusted for APACHE II score, personal history of VTE, family history of VTE, and baseline inotrope/vasopressor use, whereas hazard ratios for bleeding events were adjusted for APACHE II score. Covariates in the adjusted models were selected *a priori*. The variables in the Cox regression models were verified to meet the assumption of proportional hazards. The plots of Martingale residuals and deviance residuals did not indicate any problems with model fit.

We interpreted (2-sided) p<0.05 as statistically significant. Statistical analyses were conducted using SAS (version 9.4; Cary, USA).

## Results

### Patients

**Fig 1:**
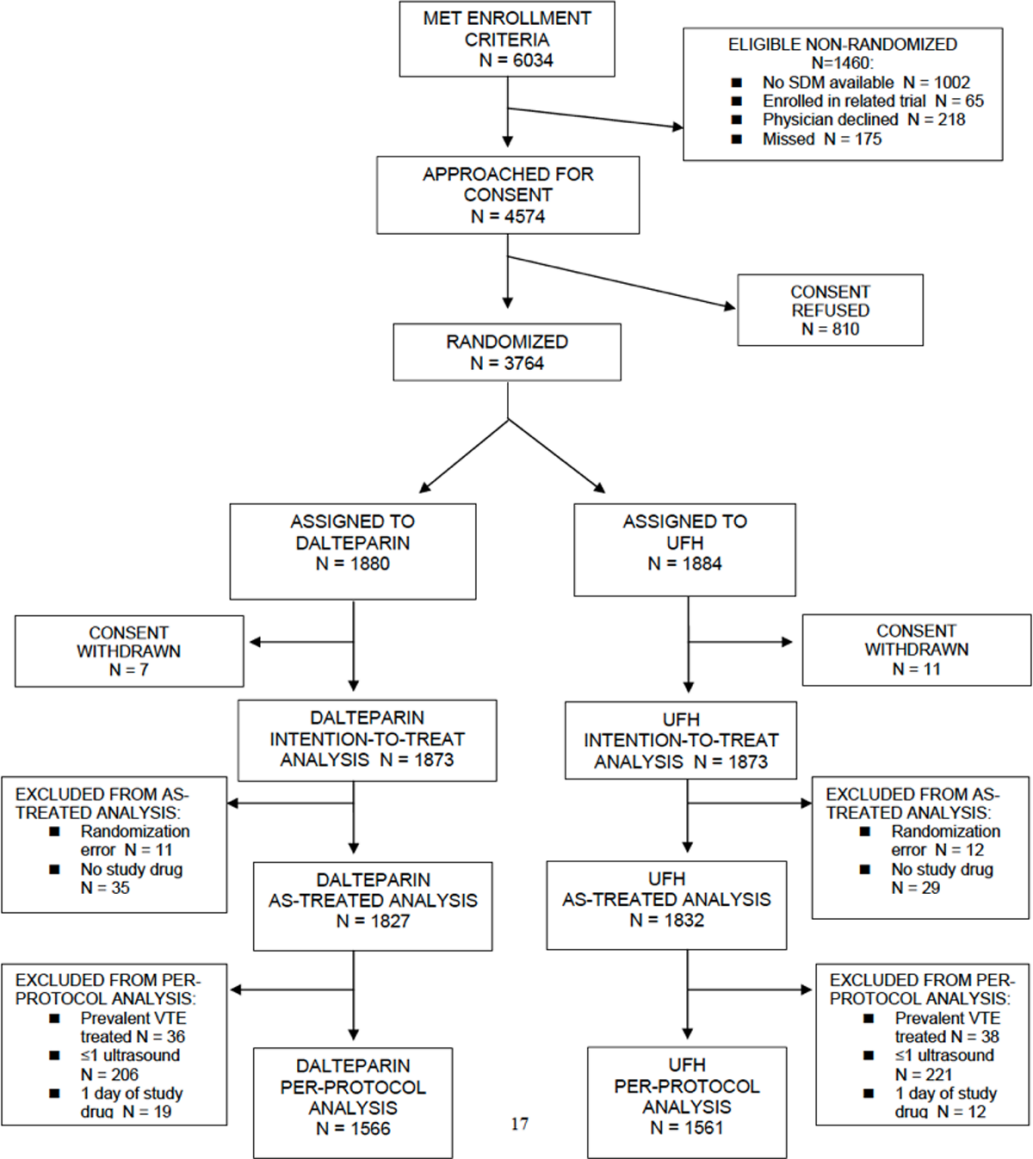
CONSORT diagram: Patient flow through the study.

Of the 6034 patients who met the enrollment criteria for PROTECT, 4574 were approached for consent (Fig 1). Consent was obtained for 3764 of these patients (82.3%). Consent was subsequently withdrawn for 18 patients. Of the 3746 patients in the intention-to-treat analysis, 1873 patients were assigned to receive dalteparin and 1873 to receive unfractionated heparin. No patients were lost to follow-up. This substudy focused on 118 patients with ESRD (mean age, 63 years; 39% female), 590 patients with ESRD or CrCl <30 mL/min (mean age, 69 years; 46% female; this group included the 118 patients with ESRD plus 472 patients not on dialysis with CrCl <30 mL/min), and 3089 patients with CrCl ≥30 mL/min. The median (IQR) duration of DVT prophylaxis was 7 (4–12) days in all patients. Additional patient characteristics are shown in Table 1.

**Table 1:** Patient characteristics.

	ESRD (N = 118)	ESRD or CrCl <30 ml/min and no dialysis (N = 590)	CrCl ≥30 ml/min (N = 3089)
**Demographics**			
Age, mean (SD)	62.6 (14.1)	68.6 (14.6)	60.1 (16.5)
Female, N (%)	46 (39.0)	270 (45.8)	1320 (42.7)
APACHE II score, mean (SD)	27.3 (7.4)	26.9 (7.3)	20.5 (7.4)
BMI, mean (SD)	28.1 (7.5)	27.3 (6.7)	28.5 (7.9)
Medical admission, N (%)	94 (79.7)	449 (76.1)	2327 (75.3)
Personal history of VTE, N (%)	8 (6.8)	20 (3.4)	99 (3.2)
Family history of VTE, N (%)	1 (0.8)	5 (0.8)	51 (1.7)
History of malignancy, N (%)	5 (4.2)	21 (3.6)	126 (4.1)
**Admitting diagnosis, N (%)**			
Cardiovascular	14 (11.9)	65 (11.0)	266 (8.6)
Respiratory	31 (26.3)	179 (30.3)	1496 (48.4)
Gastrointestinal	9 (7.6)	86 (14.6)	430 (13.9)
Renal	13 (11.0)	45 (7.6)	20 (0.6)
Neurologic	8 (6.8)	17 (2.9)	205 (6.6)
Sepsis	31 (26.3)	151 (25.6)	398 (12.9)
Metabolic	2 (1.7)	20 (3.4)	122 (3.9)
Other–medical	2 (1.7)	9 (1.5)	54 (1.7)
Other–surgical	8 (6.8)	18 (3.1)	98 (3.2)
**Life support, N (%)**			
Mechanical ventilation	94 (80.3)	513 (87.1)	2808 (90.9)
Vasopressors	57 (48.7)	349 (59.3)	1305 (42.2)
Central venous catheterization	108 (92.3)	548 (93.0)	2483 (80.4)

### Comparison of outcomes with dalteparin and UFH prophylaxis

In patients with ESRD (Table 2), there was no significant difference between those who received dalteparin and UFH in the incidence of proximal DVT (8.3% vs. 5.2%; HR 1.32, 95% CI 0.23–7.67, p = 0.76) or any VTE (10.0% vs. 6.9%; HR 2.08, 95% CI 0.39–11.17, p = 0.39). No PEs were observed in this group. There was no significant difference in major bleeds between patients who received dalteparin and UFH (5.0% vs. 8.6%; HR 0.47, 95% CI 0.11–2.08, p = 0.32).

**Table 2:** Subgroup analyses based on baseline ESRD (pre-ICU chronic dialysis).

**ESRD**	**Total (N = 118)**	**Dalteparin (N = 60)**	**UFH (N = 58)**	**Hazard Ratio† (95% CI)**	**p-value**
Proximal DVT, N (%)	8 (6.8)	5 (8.3)	3 (5.2)	1.32 (0.23–7.67)	0.76
VTE, N (%)	10 (8.5)	6 (10.0)	4 (6.9)	2.08 (0.39–11.17)	0.39
PE, N (%)	0 (0.0)	0 (0.0)	0 (0.0)	-	-
Major bleed, N (%)	8 (6.8)	3 (5.0)	5 (8.6)	0.47 (0.11–2.08)	0.32
**No ESRD**	**Total (N = 3609)**	**Dalteparin (N = 1805)**	**UFH (N = 1804)**	**Hazard Ratio† (95% CI)**	**p-value**
Proximal DVT, N (%)	197 (5.5)	91 (5.0)	106 (5.9)	0.91 (0.67–1.22)	0.52
VTE, N (%)	330 (9.1)	148 (8.2)	182 (10.1)	0.85 (0.67–1.08)	0.18
PE, N (%)	67 (1.9)	24 (1.3)	43 (2.4)	0.50 (0.29–0.86)	0.01
Major bleed, N (%)	200 (5.5)	100 (5.5)	100 (5.5)	1.03 (0.77–1.38)	0.83

Abbreviations: DVT, deep venous thrombosis; ESRD, end-stage renal disease; PE, pulmonary embolism; UFH, unfractionated heparin; VTE, venous thromboembolism.

† Hazard ratios obtained from Cox models stratified by centre and medical/surgical admission. Each model contains a term for group (baseline ESRD vs. not), treatment group (dalteparin vs. UFH), and an interaction term. For thrombotic events (proximal DVT, VTE, PE), hazard ratios are adjusted for APACHE II score, personal history of VTE, family history of VTE, and baseline inotrope/vasopressor use. For major bleed events, hazard ratios are adjusted for APACHE II score. Interaction p-values between adjusted hazard ratios for patients with ESRD and patients without ESRD were not significant (p = 0.68 for proximal DVT, p = 0.30 for VTE, and p = 0.31 for major bleeding).

In patients with severe renal dysfunction (ESRD or CrCl <30 mL/min without dialysis dependence, Table 3), there were significantly more proximal DVTs in patients who received dalteparin than UFH (7.6% vs. 3.7%; HR 0.47, 95% CI 0.11–2.08, p = 0.04). There was a non-significant increase in any VTE in the dalteparin group (10.0% vs. 6.4%; HR 1.87, 95% CI 0.96–3.63, p = 0.07). There was no significant difference in PEs (0.7% vs. 1.0%; HR 0.37, 95% CI 0.04–3.68, p = 0.39) and no significant difference in major bleeds (8.9% vs. 11.0%, HR 0.89, 95% CI 0.51–1.53, p = 0.66) between patients who received dalteparin and UFH.

**Table 3:** Subgroup analyses based on baseline severe renal dysfunction (ESRD or CrCl <30 mL/min without dialysis dependence).

**Severe renal dysfunction**	**Total (N = 590)**	**Dalteparin (N = 291)**	**UFH (N = 299)**	**Adjusted Hazard Ratio† (95% CI)**	**p-value**
Proximal DVT, N (%)	33 (5.6)	22 (7.6)	11 (3.7)	2.34 (1.03–5.34)	0.04
Any VTE, N (%)	48 (8.1)	29 (10.0)	19 (6.4)	1.87 (0.96–3.63)	0.07
Any PE, N (%)	5 (0.8)	2 (0.7)	3 (1.0)	0.37 (0.04–3.68)	0.39
Major bleed, N (%)	59 (10.0)	26 (8.9)	33 (11.0)	0.89 (0.51–1.53)	0.66
**No severe renal dysfunction**	**Total (N = 3089)**	**Dalteparin (N = 1550)**	**UFH (N = 1539)**	**Adjusted Hazard Ratio† (95% CI)**	**p-value**
Proximal DVT, N (%)	171 (5.5)	73 (4.7)	98 (6.4)	0.78 (0.56–1.08)	0.13
Any VTE, N (%)	290 (9.4)	124 (8.0)	166 (10.8)	0.77 (0.60–0.99)	0.04
Any PE, N (%)	61 (2.0)	22 (1.4)	39 (2.5)	0.50 (0.29–0.88)	0.02
Major bleed, N (%)	145 (4.7)	75 (4.8)	70 (4.5)	1.08 (0.76–1.52)	0.68

Abbreviations: DVT, deep venous thrombosis; ESRD, end-stage renal disease (defined as dialysis dependence before ICU admission); PE, pulmonary embolism; UFH, unfractionated heparin; VTE, venous thromboembolism.

† Hazard ratios obtained from Cox models stratified by centre and medical/surgical admission. Each model contains a term for group (severe renal dysfunction vs. not), treatment group (dalteparin vs. UFH), and an interaction term. For thrombotic events (proximal DVT, VTE, PE), hazard ratios are adjusted for APACHE II score, personal history of VTE, family history of VTE, and baseline inotrope/vasopressor use. For major bleed events, hazard ratios are adjusted for APACHE II score. Interaction p-values between adjusted hazard ratios for patients with baseline renal dysfunction vs. not were significant for proximal DVT (p = 0.02) and VTE (p = 0.01), but not for PE (p = 0.80) or major bleeding (p = 0.56).

When VTE and major bleeding outcomes were analyzed in subgroups defined by ESRD, the interaction p-values for the difference between all HRs were non-significant (p>0.05) (Table 2). When similar analyses were done for patients with severe renal dysfunction (ESRD or CrCl<30 mL/min without dialysis dependence), interaction p-values for the difference between the HRs for proximal DVT (p = 0.02) and VTE (p = 0.01) were significant. Dalteparin prophylaxis was associated with a higher risk for proximal DVT and any VTE in patients with severe renal dysfunction, which was statistically significant for proximal DVT, while dalteparin was associated with lower risk for these events in patients without severe renal dysfunction (i.e. patients with CrCl >30 mL/min), which was statistically significant for any VTE. All other interaction p-values were non-significant (p>0.05) (Table 3).

In a further exploratory analysis, we assessed possible imbalances in VTE risk factors (not adjusted for in the regression analysis) among patients with severe renal dysfunction who received dalteparin and UFH. We found no differences between treatment groups in mean body mass index (27.4 kg/m^2^ vs. 27.2 kg/m^2^), or in the proportion of patients with a femoral vein catheter (19.7% vs. 18.4%) or any central venous catheter (94.8% vs. 96.7%).

## Discussion

In critically ill patients with dialysis-dependent ESRD at baseline, an *a priori* subgroup analysis of data from the PROTECT trial showed no significant difference in proximal DVT, any VTE or major bleeding between VTE prophylaxis with dalteparin, 5000 IU daily, compared to UFH, 5000 IU twice-daily. In a *post hoc* analysis of patients with severe renal dysfunction, defined as baseline ESRD or CrCl <30 mL/min without dialysis dependence, there was no significant difference in major bleeding between the dalteparin and UFH groups. However, patients who received dalteparin had a non-significant increased risk of any VTE and a significant increased risk of proximal DVT.

The overall results of the PROTECT trial, which includes the patients presented in this report, showed that dalteparin, compared with UFH, had no significant difference in major bleeding (5.5% vs. 5.6%) and proximal DVT (5.1% vs. 5.8%) but a significantly lower risk of PE (1.3% vs. 2.3%) when used for VTE prophylaxis in critically ill patients[2]. The results of the present subgroup analysis are consistent with the overall findings for the safety outcome of major bleeding and support the premise that low-dose dalteparin, when administered over a 7–10 day period, appears safe for use in patients with ESRD or CrCl <30 mL/min without dialysis dependence.

However, there are some discrepant findings for the efficacy outcomes that require careful interpretation. Whereas rates of proximal DVT were not significantly different in the dalteparin and UFH groups with ESRD (8.3% vs. 5.2%, p = 0.76), there was a significantly higher rate of proximal DVT in the dalteparin than UFH group when a larger population with severe renal dysfunction (7.6% vs. 3.7%, p = 0.04) was studied. Acknowledging that the latter was a *post hoc* comparison, there are three possible explanations for these discrepant findings. First, it is possible that the findings are true and are explained by a more effective anticoagulant effect of UFH over dalteparin in patients with ESRD or CrCl <30 mL/min. Potentially supporting this premise is the observation that major bleeding events were numerically higher in the UFH than dalteparin group (11.0% vs. 8.9%, p = 0.76); however, the risks were not statistically distinguishable. Although one may therefore speculate that heparin has a greater overall anticoagulant effect compared with dalteparin, this premise is unlikely, as low-dose UFH is cleared mainly by non-renal mechanisms [5]. At the doses used in the PROTECT trial, it would be unlikely for UFH to bioaccumulate more than dalteparin, leading to more bleeds and fewer DVT events. Second, it is possible that the findings are true and are explained by a more persistent antithrombotic effect of the twice-daily dosing of UFH than the once-daily dosing of dalteparin, which may have more impact among patients with severe renal dysfunction compared to other patients. Supporting this premise is that in patients without severe renal dysfunction (i.e. those with CrCl ≥30 ml/min), rates of proximal DVT were numerically lower in the dalteparin than UFH group (4.7% vs. 6.4%, p = 0.13). Finally, the finding of more proximal DVT in the dalteparin than UFH group may be spurious and due to the play of chance. In support of this premise, the subgroup with severe renal dysfunction represented only 16% of the total PROTECT study population. Moreover, the effect of more proximal DVT in the dalteparin group was not evident in the closely related outcome of PE.

On balance, further study is required before any definitive conclusions can be made about the efficacy of dalteparin compared with UFH for the prevention of proximal DVT in patients with severe renal dysfunction. Similarly, further study is required to explore the numerically increased (albeit statistically insignificant) major bleeding events in the UFH group. Renal dysfunction results in complex hemostatic imbalances, including decreased platelet aggregation and impaired platelet adhesion (partly due to impaired function of platelet membrane glycoprotein IIb/IIIa) [17, 18]. UFH and LMWH have different effects on platelet receptor activation and platelet aggregation in vitro, so it is possible that their in vivo actions in individuals with impaired primary hemostasis also differ, leading to more bleeding with UFH [19].

We acknowledge additional potential limitations of our study. First, the number of patients studied, especially those with ESRD, was relatively small compared to the entire study sample. As a result, confidence intervals for effects that were statistically negative were wide, and effect estimates may change in future trials with more patients and events. Although this is the largest study, to our knowledge, of patients with ESRD who received LMWH prophylaxis, additional research is warranted. Second, the mean duration of anticoagulant prophylaxis was 7 days, so our findings may not be applicable to patients who receive a considerably longer duration of prophylaxis. Third, the study did not consider the efficacy and safety of LMWHs other than dalteparin.

Our findings are relevant to clinical practice as they further challenge the premise that LMWHs should be avoided in patients with renal dysfunction because of presumed bioaccumulation and the potential for an increase in bleeding. We acknowledge the inconsistent finding for the outcome of DVT in the groups with ESRD compared to the larger subgroup of severe renal dysfunction (ESRD or CrCl<30 mL/min without dialysis dependence); we believe that this finding should be interpreted with caution and warrants further study.

In critically ill patients with ESRD and those with severe renal dysfunction more broadly defined, there was no significant difference in any VTE or major bleeding between UFH and dalteparin. Patients with ESRD or CrCl <30 mL/min without dialysis dependence who received dalteparin had more proximal DVTs than those who received UFH; this finding did not hold in patients with ESRD alone. This discrepant finding merits further investigation.

## Supporting information

S1 Appendix List of participating hospitals. (DOCX)

S2 Appendix PROTECT study protocol. (PDF)

S3 Appendix CONSORT 2010 checklist. (PDF)
