# Supplementary material for: Low-molecular-weight heparin venous thromboprophylaxis in critically ill patients with renal dysfunction: A subgroup analysis of the PROTECT trial
Source: PLoS One. 2018 Jun 1;13(6):e0198285. doi: 10.1371/journal.pone.0198285 (PMC5983525; doi:10.1371/journal.pone.0198285)
Supplement: S1 Appendix — (DOCX) [file pone.0198285.s001.docx]

**S1 Appendix: List of Participating Hospitals**

St Joseph’s Healthcare, Hamilton; Capital Health Queen Elizabeth II Health Science Center, Halifax; Hamilton Health Sciences, Hamilton; University Health Network, Toronto; Hôpital de l'Enfant-Jésus, Quebec City; Charles LeMoyne Hospital, Montreal; Ottawa Hospital, Ottawa; Maisonneuve Rosemont Hospital, Montreal; Hôpital du Sacré-Coeur de Montréal, Montreal; Mount Sinai Hospital, Toronto; Sunnybrook Hospital, Toronto; St Michael’s Hospital, Toronto; Kingston General Hospital, Kingston; Royal Columbian Hospital, Westminster; Vancouver General Hospital, Vancouver; St. Paul’s Hospital, Vancouver; Calgary University Foothills Hospital, Calgary; Royal Alexandra Hospital, Edmonton; Laval Hospital, Quebec City; University of Alberta, Edmonton; Calgary University Peter Lougheed Hospital, Calgary; Montreal General Hospital, Montreal; Vancouver Island Health Authority, Vancouver; Sherbrooke University Hospital and Centre de Recherche Clinique Étienne-Le Bel, Sherbrooke; Royal Victoria Hospital, Montreal; Guelph General Hospital, Guelph; Grand River Hospital, Kitchener; St Boniface Hospital, Winnipeg; Lakeridge Health, Oshawa; Surrey Memorial Hospital, Surrey; London Health Sciences Center, London; Alfred Hospital, Melbourne; Royal North Shore Hospital, Sydney; Royal Melbourne Hospital, Melbourne; Austin Hospital, Melbourne; Box Hill Hospital, Melbourne; Frankston Hospital, Frankston; Monash Medical Centre, Melbourne; Nepean Hospital, Sydney; Royal Adelaide Hospital, Adelaide; Royal Prince Alfred Hospital, Camperdown; Bendigo Health Care, Bendigo; Blacktown Hospital, Blacktown; Flinders Medical Center, Adelaide; Geelong Hospital, Geelong; Dandenong Hospital, Dandenong; Wollongong Hospital, Wollongong; Lyell McEwin Hospital, Adelaide; Santa Casa Hospital, Porto Allegre; Moinhos de Vento Hospital, Porto Alegre; Pró Cardíaco Hospital, PROCEP, Rio de Janeiro; Hospital Coracao Research Institute HCor, São Paulo; Hospital São Paulo, São Paulo; King Faisal Specialist Hospital and Research Center, Jeddah; King Saud Bin Abdulaziz University for Health Sciences, Riyadh; King Abdulaziz University Hospital, Jeddah; Riyadh Military Hospital, Riyadh; King Fahad Medical City Hospital, Riyadh; Rhode Island Hospital, Providence; Mayo Clinic, Rochester; St John’s Mercy Medical Center, St Louis; University of Texas MD Anderson Cancer Center, Houston; King's College London, Guy's & St Thomas’ Hospital, London; St Joseph’s Healthcare, Hamilton; Capital Health Queen Elizabeth II Health Science Center, Halifax; Hamilton Health Sciences, Hamilton; University Health Network, Toronto; Hôpital de l'Enfant-Jésus, Quebec City; Charles LeMoyne Hospital, Montreal; Ottawa Hospital, Ottawa; Maisonneuve Rosemont Hospital, Montreal; Hôpital du Sacré-Coeur de Montréal, Montreal; Mount Sinai Hospital, Toronto; Sunnybrook Hospital, Toronto; St Michael’s Hospital, Toronto; Kingston General Hospital, Kingston; Royal Columbian Hospital, Westminster; Vancouver General Hospital, Vancouver; St. Paul’s Hospital, Vancouver; Calgary University Foothills Hospital, Calgary; Royal Alexandra Hospital, Edmonton; Laval Hospital, Quebec City; University of Alberta, Edmonton; Calgary University Peter Lougheed Hospital, Calgary; Montreal General Hospital, Montreal; Vancouver Island Health Authority, Vancouver; Sherbrooke University Hospital and Centre de Recherche Clinique Étienne-Le Bel, Sherbrooke; Royal Victoria Hospital, Montreal; Guelph General Hospital, Guelph; Grand River Hospital, Kitchener; St Boniface Hospital, Winnipeg; Lakeridge Health, Oshawa; Surrey Memorial Hospital, Surrey; London Health Sciences Center, London; Alfred Hospital, Melbourne; Royal North Shore Hospital, Sydney; Royal Melbourne Hospital, Melbourne; Austin Hospital, Melbourne; Box Hill Hospital, Melbourne; Frankston Hospital, Frankston; Monash Medical Centre, Melbourne; Nepean Hospital, Sydney; Royal Adelaide Hospital, Adelaide; Royal Prince Alfred Hospital, Camperdown; Bendigo Health Care, Bendigo; Blacktown Hospital, Blacktown; Flinders Medical Center, Adelaide; Geelong Hospital, Geelong; Dandenong Hospital, Dandenong; Wollongong Hospital, Wollongong; Lyell McEwin Hospital, Adelaide; Santa Casa Hospital, Porto Allegre; Moinhos de Vento Hospital, Porto Alegre; Pró Cardíaco Hospital, PROCEP, Rio de Janeiro; Hospital Coracao Research Institute HCor, São Paulo; Hospital São Paulo, São Paulo; King Faisal Specialist Hospital and Research Center, Jeddah; King Saud Bin Abdulaziz University for Health Sciences, Riyadh; King Abdulaziz University Hospital, Jeddah; Riyadh Military Hospital, Riyadh; King Fahad Medical City Hospital, Riyadh; Rhode Island Hospital, Providence; Mayo Clinic, Rochester; St John’s Mercy Medical Center, St Louis; University of Texas MD Anderson Cancer Center, Houston; King's College London, Guy's & St Thomas’ Hospital, London
