# Supplementary material for: Low-molecular-weight heparin venous thromboprophylaxis in critically ill patients with renal dysfunction: A subgroup analysis of the PROTECT trial
Source: PLoS One. 2018 Jun 1;13(6):e0198285. doi: 10.1371/journal.pone.0198285 (PMC5983525; doi:10.1371/journal.pone.0198285)
Supplement: S2 Appendix — (PDF) [file pone.0198285.s002.pdf]

Protocol

This trial protocol has been published in:

The PROTECT Investigators for the Canadian Critical Care Trials Group and the Australian and New Zealand Intensive Care Society Clinical Trials Group. Dalteparin versus unfractionated heparin in critically ill patients. N Engl J Med 2011;364:1305-14. DOI: 10.1056/NEJMoa1014475.

Cook D, Meade M, Guyatt G, Walter SD, Heels-Ansdell D, Geerts W, Warkentin TE, Cooper DJ, Zytaruk N, Vallance S, Berwanger O, Rocha M, Qushmaq I, Crowther M. PROphylaxis for ThromboEmbolism in Critical Care Trial protocol and analysis plan. J Crit Care. 2011 Apr;26(2):223.e1-9. doi: 10.1016/j.jcrc.2011.02.010.

PROTECT (Prophylaxis for ThromboEmbolism in Critical Care Trial)
Protocol and Analysis Plan

D Cook [1,2]
M Meade [1,2]
G Guyatt [1,2]
SD Walter [2]
D Heels-Ansdell [2]
W Geerts [3]
TE Warkentin [1,4]
DJ Cooper [5,6]
N Zytaruk [2]
S Vallance [5]
O Berwanger [7]
M Rocha [8]
I Qushmaq [9]
M Crowther [1,4]

For the PROTECT Investigators, the Canadian Critical Care Trials Group and the Australian and New Zealand Intensive Care Society Clinical Trials Group

Departments of Medicine[1] Clinical Epidemiology & Biostatistics[2] & Pathology & Molecular Medicine[4], McMaster University, Hamilton, Canada
Department of Medicine, University of Toronto, Toronto, Canada[3]
Department of Intensive Care Medicine, The Alfred[5], Department of Epidemiology and Preventive Medicine, Monash University[6], Melbourne, Australia
Research Institute, Hospital do Coracao, Sao Paolo, Brazil[7]
Pavilhao Pereira Filho, Irmandade Santa Casa de Misericordia, Porte Allegre, Brazil[8]
Department of Critical Care, King Faisal Hospital, Jeddah, Saudi Arabia[9]

Correspondence:
DJ Cook, Departments of Medicine & Epidemiology and Biostatistics,
McMaster University Health Sciences Center, Room 2C11,
1200 Main Street West, Hamilton, Ontario, Canada, L8N 3Z5
Phone: 905 525 9140 x 23163, Fax: 905 524 3841, Email: debcook@mcmaster.ca

Word Count: 4542, not including subtitles, tables, legends and references
Abstract Word Count: 314

NOTE: This manuscript is currently in press at the Journal of Critical Care

Abstract:

Background: This manuscript reports the preparatory studies, as well as the design, implementation and *a priori* analysis plans of PROTECT prior to dissemination of results. PROphylaxis for ThromboEmbolism in Critical Care Trial (NCT00182143) is a randomized, stratified, concealed international trial comparing subcutaneous injection of unfractionated heparin 5,000 IU, or the low molecular weight (LMWH) dalteparin 5,000 IU once daily plus once daily placebo for the duration of the intensive care unit stay.

Methods: The objective of PROTECT is to examine, among medical-surgical critically ill patients, the effect of the LMWH versus heparin on the primary outcome of proximal leg deep vein thrombosis (DVT), and the following secondary outcomes: DVT elsewhere, pulmonary embolism, any venous thromboembolism (DVT or PE), venous thromboembolism or death, bleeding and heparin-induced thrombocytopenia (HIT). Patients are followed to death or hospital discharge. VTE events were included after ICU discharge. All patients, families, clinicians, research personnel, and the trial biostatistician are blind to allocation.

Results: We describe the pilot work, large trial methodology and implementation methods, as well as the analytic plan. Patient recruitment is complete but 2 patients remain in hospital. The rigorous design of PROTECT suggests that the risk of systematic error will be low. The sample size suggests that the risk of random error will be low. PROTECT will be the largest investigator-initiated, peer-review funded thromboprophylaxis trial in critical care in the world.

Conclusions: If PROTECT shows that LMWH is more effective than UFH, this trial will change practice in that LWMH may be the anticoagulant thromboprophylaxis of choice for this population. If the results show that UFH is as effective or more effective than LMWH, intensivists in many parts of the world may continue to use UFH while those currently using LMWH may reconsider and change to use UFH. Unfavourable consequences of major bleeding, HIT, drug availability and the costs of complications will also factor into such decisions.

Venous Thromboembolism in the ICU

Changes in blood coagulation, inflammation and the host immune response are intricately linked and interdependent, rendering the development of venous thromboembolism (VTE) an important complication of critical illness. Patients in the intensive care unit (ICU) have an increased risk of both deep vein thrombosis (DVT) and pulmonary embolism (PE), due to their complex acute and chronic illnesses, need for life support, immobility due to sedation, analgesia and paralysis, and procedures such as surgery and central venous catheterization [1].

Although DVT has potentially serious consequences, it is usually unrecognized in the ICU. Neither structured physical examination [2] nor thrombophilia markers [3] can help to identify DVT in this setting. Studies show that 10% [4,5] to 100% [6,7] of DVTs in critically ill patients found by ultrasound screening were not detected on physical examination. When compared with patients who did not have DVT, those with DVT are reported to have a longer duration of mechanical ventilation (p=0.02), longer duration of ICU stay (p=0.005), longer duration of hospital stay (p<0.001) and higher hospital mortality (p=0.04) [5].

Among critically ill patients, clinically unsuspected PE is also a problem. Mechanically ventilated patients with sudden episodes of hypotension, tachycardia, or hypoxemia may have undetected PE [6]. In one study, 13 of 34 (38%) of ICU patients with known DVT and no symptoms of PE had PE diagnosed by ventilation-perfusion scans [7]. PE may also contribute to difficulty weaning patients from mechanical ventilation [8]. In ICU patients with impaired cardiopulmonary reserve, even a small PE might have severe or fatal consequences [9]. In another study, PE was unsuspected in 14 of 20 (70%) patients who died from PE [10]. In a 25-year longitudinal study, 9% of patients had PE at autopsy; the antemortem diagnosis of PE was missed in 84% of these patients [11]. VTE remains one of the commonest unrecognized clinical entities in the intensive care unit found at autopsy [12].

Anticoagulant Thromboprophylaxis

In 2 early meta-analyses of trials enrolling over 8,000 general surgery patients, prophylactic subcutaneous unfractionated heparin (UFH) resulted in a 60-70% relative risk reduction for both DVT and fatal PE [13,14]. Given the high prevalence and potential morbidity and mortality associated with VTE, thromboprophylaxis should be used during critical illness [15]. However, only 4 randomized trials of thromboprophylaxis in medical-surgical ICU patients have been published; 2 tested anticoagulant thromboprophylaxis versus placebo [16,17], and 2 tested active comparators [18,19].

In one trial, 199 medical-surgical ICU patients were randomized to subcutaneous UFH 5,000 IU twice daily or placebo [16]. Using serial fibrinogen leg scanning for 5 days, DVT rates were 13% in the UFH group and 29% in the placebo group (relative risk reduction [RRR] 0.55, p<0.05). Bleeding, PE and mortality were not reported. Subsequent demonstration that leg scanning is not a reliable DVT diagnostic test confounds interpretation of this study [20]. In the second trial, Fraisse and colleagues randomized 223 patients with an acute exacerbation of COPD requiring mechanical

ventilation to the low molecular weight heparin (LMWH) nadroparin (3,800 anti-Xa U for 45-70 kg or 5,700 anti-Xa U for 71-110 kg) once daily or placebo [17]. Patients had weekly duplex ultrasounds, and venography was attempted at 21 days or if ultrasound results were positive or non-diagnostic. Only 169 patients were evaluable (84 in the nadroparin group and 85 in the placebo group. In the nadroparin group, 16% developed DVT and 28% did in the placebo group (RRR 0.45, $p<0.05$). Trends toward increased bleeding (25 vs 18 patients, $p<0.05$) and major bleeding (6 vs 3 patients, p=NS) were observed in patients receiving nadroparin. PE was not reported; 8 patients in each group died.

Of the 2 trials comparing LMWH with UFH, in the first, patients were randomized to enoxaparin 40 mg daily vs UFH 5,000 U twice daily in 156 critically ill surgical patients undergoing major surgery [18]. Doppler ultrasound between postoperative days 5-7 detected DVT in 1.2% in the enoxaparin group and 2.7% in the UFH group (RRR 0.55, p=NS). There were significantly more bleeding events in the UFH group (18 vs 8, $p=0.01$), but there was no difference in mortality (6 vs 9 patients, $P<0.05$). PE was not reported. The second trial enrolled 1,994 patients with sepsis, who during a 4 day infusion of activated drotecogin alfa [21], were allocated in a 2:1:1 ratio to placebo, or UFH 5,000 U bid, or enoxaparin 40 mg daily. Compression ultrasonography was performed between days 4-6 and if DVT was clinically suspected. During the drotecogin infusion, DVT was found in 4.4% of patients receiving placebo, in 5.1% of those on UFH, and in 4.0% of those on enoxaparin [19] (RRR 0.22 for enoxaparin vs UFH, $p<0.05$), while one patient receiving UFH developed PE.

While LMWH appears significantly more effective than UFH at preventing venographically diagnosed DVT in trauma patients [22], its potency may translate into a higher rate of bleeding. UFH is the commonest thromboprophylactic drug for medical-surgical ICU patients, as documented in self-reported surveys [23,24] and observational studies [25,26,27,28]. UFH was also the commonest thromboprophylactic anticoagulant in the United States, documented in a 15,000 patient international registry [29]. In another 35,000 patient international registry of non-critically ill patients, LMWH was more often used than UFH [30].

An editorialist previously referred to the medical-surgical ICU as ‘the last frontier for VTE prophylaxis‘ [31]. In another editorial about choice of VTE prophylaxis for medical patients, Lederle stated that ‘for now, clinicians must make their own decisions’ [32]. The Agency for Health Care Policy Research Evidence Report & Technology Assessment document on Prevention of Venous Thromboembolism called for a large randomized trial comparing the most common methods of VTE prophylaxis, identifying DVT by routine screening [33].

The objective of this trial is to examine, among medical-surgical critically ill patients, the effect of the LMWH dalteparin versus UFH on the primary outcome of proximal leg DVT, as well as the following secondary outcomes: venous thrombosis elsewhere, PE, VTE, VTE or death, bleeding and heparin-induced thrombocytopenia (HIT).

PROTECT Pilot Trial

Before embarking on a large clinical trial, we first conducted the PROTECT (PROphylaxis for ThromboEmbolism in Critical Care Trial) Pilot trial, enrolling 128 patients in 13 centers in Canada and Australia [34]. This study randomized patients to dalteparin 5,000 IU once daily or unfractionated heparin 5,000 IU twice daily and followed them with serial proximal ultrasonography twice weekly. The feasibility objectives of the PROTECT Pilot were to assess: 1) timely enrolment and complete, blinded study drug administration, 2) LMWH bioaccumulation in renal insufficiency, 3) twice weekly leg ultrasounds, and 4) recruitment rates.

We found: 1) Timely, complete study drug administration occurred for 98% of scheduled doses; every dose was blinded. 2) No LWMH bioaccumulation was observed as measured by serial anti-Xa levels. 3) The first ultrasound was performed within 72h for 98% of patients; subsequent ultrasounds occurred as scheduled without exception. 4) The estimated recruitment for the large trial was based on doubling the recruitment in our Pilot (2 patients/month/center to 4-5 patients/month/center) after modification of 2 exclusion criteria as per the rationale below. We analyzed outcomes on all enrolled patients rather than in the 2 intervention groups to avoid inappropriate interpretation of differences found in small randomized trials [35]. Proximal lower limb DVT occurred in 8.6% of patients overall [34].

Improvements to the Main PROTECT Protocol

Over 9 months of screening for the PROTECT Pilot, we excluded 575 patients and randomized 86 patients. The 6 most frequent reasons for exclusion were: 1) creatinine clearance <30ml/min/1.73m^2 (193, 34% of reasons), 2) therapeutic anticoagulation (123, 21%), 3) hemorrhage on ICU admission (119, 21%), 4) platelet count <100 x 10^9/L (122, 21%), 5) coagulopathy (71, 12%), and 6) >2 doses of heparin in ICU before randomization (62, 11%). The main 2 clinical exclusion criteria that were modifiable were renal dysfunction and the platelet count threshold. Based on these PILOT findings, we made 2 changes to the protocol. First, we reduced the platelet count threshold for excluding patients to <75 x 10^9/L, reflecting practice. Second, we conducted DIRECT (Dalteparin's Influence on REnal Insufficiency in Critical Care Trial) to determine whether bioaccumulation of prophylactic doses of LMWH occurs in patients with creatinine clearance <30ml/min/1.73m^2. We found, that bioaccumulation of dalteparin did not occur among 138 ICU patients with renal insufficiency [36]. Therefore, we enrolled patients in PROTECT irrespective of their renal function. Although stringent exclusion criteria chosen deliberately for the Pilot trial relative to use of study drugs in practice increased the perceived safety of the Pilot, we reasoned that changing the exclusion criteria for the larger trial would enhance the applicability of the ultimate trial results without adversely affecting safety [37].

In summary, analysis of the PROTECT Pilot exclusion criteria informed the design of, and enhanced the generalizabilty of, the larger PROTECT trial, and suggested that a large trial was feasible. This was an internal pilot and enrolled patients were included in the main trial report.

Methods for the Main Trial

PROTECT (PROphylaxis for ThromboEmbolism in Critical Care Trial) (NCT00182143) is a concealed, randomized, stratified, blinded multicenter trial. Patients are considered for enrolment if they are ≥18 years of age, weigh >45 kg, and are expected to remain in ICU at least 72 hours. Exclusion criteria are admission diagnoses of trauma, neurosurgery, orthopedic surgery, uncontrolled hypertension (systolic blood pressure ≥180 mmHg or diastolic blood pressure ≥110 mmHg) for at least 12 hours, major bleeding within the last week unless definitively treated, hemorrhagic stroke, coagulopathy (international normalized ratio (INR) >2 times upper limit of normal, or activated partial thromboplastin time (aPTT) >2 times the upper limit of normal, severe thrombocytopenia (platelet count < 75 x 10^9/L), need for therapeutic anticoagulation, receipt of at least 72 hours of any dose of heparin, contraindication to heparin, blood products, or pork products, pregnancy, limitation of life support, and current enrolment in a related trial. Research coordinators obtain informed consent from substitute decision makers or eligible patients.

To ensure concealed allocation, pharmacists randomize patients using a computerized randomization system with undisclosed variable block sizes, stratified by center, and medical versus surgical admission status. Allocation concealment is ensured by access via a password-protected website or voice-activated telephone system. Research pharmacists prepare identical syringes for twice daily subcutaneous injection of UFH 5,000 IU, or dalteparin 5,000 IU once daily plus once daily placebo injection, which are administered by bedside nurses for the duration of ICU stay. All patients, families, clinicians, research personnel, ultrasonographers, outcome adjudicators and the trial biostatistician are blind to allocation for the duration of the trial.

If therapeutic anticoagulation is needed for any reason, study drug is withheld and restarted if treatment is no longer needed. If therapeutic anticoagulation is needed for the entire ICU stay, study drug is permanently discontinued but the patient remains in the trial with mandatory ultrasound screening and follow-up. Anti-embolic stockings or pneumatic compression devices are used if patients meet pre-specified criteria for active bleeding or high risk of bleeding. If the platelet count decreases to less than 50 x 10^9/L, if there is an unexplained platelet count decrease to less than 50% of baseline, or other suspicion for HIT, heparin is stopped, mechanical prophylaxis or a HIT-safe anticoagulant are started [38], and local heparin-dependent antibody test is performed using a commercially-available ELISA PF4 assay. Research coordinators also send blood to the Central Reference Laboratory at McMaster University for a serotonin-release assay which defines HIT in this trial [39]. All aspects of care and management of trial outcomes are otherwise at the ICU team's discretion.

Research coordinators collect data daily in ICU regarding life support, diagnostic tests, drugs, devices, events, and exposures that modify risk of, or define, thrombotic or bleeding events. For thrombotic events, research coordinators collect all clinical notes and diagnostic tests. For bleeding events, research coordinators document site, duration, severity, and consequences (e.g., transfusions) of bleeding using an instrument validated in the ICU [40]. Patients are followed to hospital discharge to

document vital status and VTE events. Patients are censored at 100 days; however, any VTE events identified after ICU discharge are included.

Thrombotic and Bleeding Outcomes

The primary outcome is incident proximal leg DVT, defined as detected 3 or more days post-randomization. Certified ultrasonographers perform twice weekly bilateral proximal leg venous ultrasounds, and if DVT is clinically suspected. The venous system is interrogated at 1 centimeter intervals, documenting compressibility at the following 6 sites: common femoral, proximal superficial femoral, mid superficial femoral, distal superficial femoral, popliteal, and trifurcation. We define DVT if there is a partially or completely incompressible venous segment. Venous wall thickening is not considered diagnostic of DVT. If a venous segment is not well visualized and never is well visualized on subsequent ultrasounds, the test is considered indeterminate; such events are recorded but are not considered trial outcomes. Ultrasonographers may scan the distal leg veins at their discretion.

We define prevalent DVT as documented on the first ultrasound, reflecting a baseline characteristic, not plausibly related to the study drug. Such patients are included in the main analysis but a prevalent thrombosis is not considered a primary outcome.

DVTs are considered chronic if a prior test reveals evidence of thrombus in the same or contiguous venous segment. DVTs and other VTE events are labeled as incident if they occur more than 72 hours after randomization. We define a thrombus as catheter-related if a catheter had been in situ in the same or a contiguous venous segment within 72 hours of the diagnosis.

We define PE as: definite PE (characteristic intraluminal filling defect on CT chest, high probability VQ scan or detected at autopsy); probable PE (moderate-high pretest probability [high clinical suspicion] and no test or a non-diagnostic test); possible PE (low pre-test probability (low clinical suspicion] and a non-diagnostic test); or no PE (negative or normal test without reference to pretest probability).

We define major bleeding events as overt and classified by site (e.g., gastrointestinal, intracranial), and by consequences ($\geq$ 2 units of packed red blood cells, need for surgery or death). Minor bleeds include overt bleeds not satisfying major criteria (e.g., injection site hematoma).

If clinicians suspect any VTE event, they perform tests as clinically indicated.

Pilot Studies To Adjudicate Thrombotic and Bleeding Outcomes

A previous ultrasound reliability study documented excellent agreement between 2 ultrasonographers blinded to each other's reading [41]. We also conducted formal calibration exercises for thrombotic and bleeding events by 4 adjudicators blinded to study drug and each others' assessments. We refined the adjudication guidelines and documented excellent agreement for leg DVTs [42], non-leg DVTs [43], pulmonary embolism [44] and bleeding [45] (kappa 1.0, 0.71, 0.82, 0.81, respectively). Since then,

we are randomly allocating all events to adjudication teams for duplicate (venous thrombotic and bleeding events) or quadruplicate (for PE) blinded review.

Serious Adverse Events (SAEs)

Vulnerable populations need research oversight to ensure that their safety, rights and well being are protected, and to ensure public trust [46]. Before starting this trial which compares 2 commonly used drugs, we outlined the SAEs we planned to identify and report in the protocol, for review by the Data Monitoring Committee and local Research Ethics Boards. We labeled the most concerning and expected serious adverse events as primary outcomes (e.g., DVT), or secondary outcomes (e.g., PE or HIT), precluding the need for duplicate labeling of such events as SAEs. The Data Monitoring Committee is reviewing SAEs at the time of each of the 2 pre-specified interim analyses, blinded to allocation, with the knowledge of the number of events and patients in each arm enrolled across all centers, in the context of other outcomes and with awareness of any new literature that emerges over the course of the trial [47].

Trial Management

To ensure protocol adherence and data quality, research coordinators, study pharmacists and ultrasonographers initially attended study-specific training sessions. Initial resources prepared by the Methods Center included procedure manuals, standard operating procedures, slide sets and a study website. Throughout the trial, participating centers also educate rotating ICU residents and hold periodic nursing in-services. The Methods Center is validating data throughout the recruitment period, and Methods Center staff liaises with centers frequently to ensure protocol adherence and to clarify data as necessary. The biostatistician is evaluating data integrity by central statistical monitoring [48,49]. Quality control reports provide structured audit and feedback on site-specific performance. Ongoing general feedback is provided at investigators' meetings and in periodic newsletters. The Methods Center prepares quarterly reports for the Steering Committee summarizing screening, randomization, protocol adherence, data accuracy, and completeness.

Research Oversight

Research Ethics Boards approved the protocol in all participating centers. The independent PROTECT Data Monitoring Committee is comprised of 3 clinician scientists and 1 biostatistician, who adopted a modified DAMOCLES Charter [50] outlining their roles, responsibilities, and reporting relationships. The trial biostatistician provides the Data Monitoring Committee with blinded reports regarding protocol adherence (e.g., randomization, crossovers, drug administration), indicators of trial management (e.g. enrolment, consent and data accuracy and completion rates), efficacy and safety reports including SAEs, and the 2 interim and final analyses.

Analyses

Sample Size Estimation

We estimated an 8% proximal DVT rate for patients randomized to UFH [5]. From randomized trials in other settings, we estimated the relative risk reduction (RRR) associated with LMWH compared with UFH of 30%. To detect a 30% RRR with a power of 0.8 and a 2-sided alpha of 0.05, a sample size of 1,809 patients per group

(total 3,618) was estimated. We plan to enroll 3,700 patients to account for potential errors in eligibility assessment or randomization, and consent withdrawal.

Statistical Analysis
The trial biostatistician is blinded to study group for interim, secondary and final analyses until the database is locked. Analyses will be conducted according to the pre-specified plan outlined herein. Patients will be analyzed in the group to which they were randomized, according to the intention to treat principle. All tests will be 2-sided.

Main Results
The main analysis will be an unadjusted survival analysis taking into account the 2 stratified randomized variables of medical versus surgical admission diagnosis, and center. This time-to-event approach will use all information up to the time of censoring such that patients remain in the denominator and contribute information while they are at risk. The assumption for this analysis is that censoring is uninformative. Our approach to adjustment is summarized in Table 1.

The primary outcome is objectively confirmed proximal leg DVT. The secondary outcomes include distal leg DVT, non-leg DVT, PE, all VTE, VTE or death, major bleeding, minor bleeding and HIT. Other outcomes are severe thrombocytopenia [$<50 \times 10^9$/L], and serious adverse events.

The composite outcome of VTE reflects the overall disease entity. The composite of VTE or death addresses the competing risk problem in that if a patient dies, she cannot have a VTE event --unless she dies of a PE, which we will seldom know since we underdiagnose PE in the ICU setting. This analysis does not assume that the censoring of deaths is uninformative; rather it allows for the possibility that deaths and VTEs could have correlated risks.

We will also record all catheter-related thromboses and indeterminate DVTs. However, we will only report indeterminate DVTs if the patient did not have any other DVTs.

The tertiary outcomes are duration of ventilation, duration of ICU stay, duration of hospital stay, ICU mortality, and hospital mortality. For the first 3 tertiary outcomes, we will compare the 2 arms using a non-parametric approach to compare median durations because the distributions of these variables are usually skewed. We will also compare survival distributions using a log rank test. For the time-to-event analyses, we will calculate hazard ratios and associated 95% confidence intervals using Cox regression analysis.

Although we believe that baseline imbalances are unlikely in this large trial, we will also conduct adjusted analyses for the VTE outcomes and bleeding. We will adjust based on the principles of a) few variables, b) only baseline variables, and c) only variables that have been shown in prior multivariable analyses to influence the outcome rate. For VTE events, these include: a) APACHE II score, b) personal or family history of VTE, c) need for inotropes or vasopressors and d) end stage dialysis-dependent renal failure [5]. For bleeding outcomes, the adjustment variables include: a) APACHE II score, and b) end

stage dialysis-dependent renal failure [36]. To the extent that the adjusted and unadjusted analyses yield similar results, inferences about the outcomes will be strengthened.

The second analysis will compare the proportion of patients in the 2 groups with the primary and secondary outcomes using the Mantel-Haenszel Chi square test or the Fisher exact test. We will calculate the relative risks and 95% confidence intervals. To the extent that this secondary analysis and the main unadjusted and adjusted analyses yield similar results, inferences about the outcomes will be strengthened. If there is a clinically important and/or statistically significant difference between groups, and if otherwise appropriate, we will calculate metrics such as the number needed to administer prophylaxis or number needed to harm.

An intention-to-treat analysis including all randomized patients will exclude those who had early consent withdrawal. The *a priori* modified intention-to-treat (effectiveness, or as-treated) analysis will include all randomized patients, excluding without bias [51] those patients who a) were randomized in error, b) had early consent withdrawal or c) never received study drug. These patients excluded without bias will also be adjudicated through a duplicate independent review process blinded to study drug, ensuring allocation could have no influence on the decision. The *a priori* per-protocol (or efficacy) analysis including all patients who have at least 2 days of study drug, at least 2 technically adequate, legitimate VTE tests, and who do not have a prevalent VTE that is treated (this will be defined as a VTE diagnosed up to 3 days post randomization, having treatment started within 3 days and continuing for at least 7 days). The efficacy population will contribute to the study question in that they have sufficient drug exposure that the outcome could be influenced by the study drug, and there is no need to assume no thrombosis in the absence of a second VTE test. Legitimate VTE tests include compression ultrasound, contrast venography, contrast-enhanced CT scans, VQ scan, and autopsy results. Legitimate VTE tests do not include echocardiography, clinical impressions or clinical death summaries.

Sensitivity Analyses

1) For the main analysis, we will label DVTs and other VTE events as incident if they occur more than 72 hours after randomization; these outcomes are the focus of the main analyses. In the first sensitivity analysis, we will consider any non-leg DVT or PE as incident if they occur on day 2 post randomization or beyond.

2) For the main analysis, we will consider all outcomes as defined in the foregoing sections. In the second sensitivity analysis, we will consider only VTE events which were clinically suspected by the ICU team. A VTE outcome will be considered clinically suspected if a test is performed to identify it, prompted by a clinical suspicion, and the finding is confirmed. A VTE outcome will be considered incidental if it is found on a test which likely or expressly was conducted to diagnose another condition. Examples include an incidental internal jugular vein thrombus identified on ultrasound of the thyroid, or an incidental PE identified on computed tomography of the abdomen to rule out intra-abdominal abscess.

Subgroup Analyses
We will conduct 3 subgroup analyses using a survival analysis approach to evaluate the treatment effect of LMWH versus UFH. We will evaluate any difference in treatment effect using a test for interaction, comparing the hazard ratios for the subgroups.

1) We will conduct subgroup analyses among medical and surgical patients as per the baseline stratification classification. Our first hypothesis is that if overall, LMWH is associated with a lower VTE rate (primary outcome of DVT, PE, any VTE) than UFH, the effect will be exaggerated among surgical patients because of their higher baseline risk of VTE. Our second hypothesis is that if overall, LMWH is associated with a higher bleeding rate than UFH, the effect will be exaggerated among surgical patients because of their higher baseline risk of bleeding due to more potential bleeding sites.

2) We will conduct subgroup analyses on patients requiring, and not requiring inotropes or vasopressors at baseline. Our first hypothesis is that if overall, LMWH is associated with a lower VTE rate (primary outcome of DVT, PE, any VTE) than UFH, the effect will be attenuated among patients receiving inotropes or vasopressors because of poor absorption and decreased bioavailability of subcutaneous heparin and LMWH in these patients. Our second hypothesis is that if overall, LMWH is associated with a higher bleeding rate than UFH, the effect will be exaggerated among patients requiring inotropes or vasopressor because of their higher baseline risk of bleeding due to thrombocytopenia and coagulopathy.

3) We will conduct subgroup analyses on patients with end-stage dialysis-dependent renal failure or not at baseline. Our first hypothesis is that if overall, LMWH is associated with a lower VTE rate (primary outcome of DVT, PE, any VTE) than UFH, the effect will be exaggerated among end-stage dialysis-dependent patients because of their higher baseline risk of VTE. Our second hypothesis is that if overall, LMWH is associated with a higher bleeding rate than UFH, the effect will be exaggerated among patients with end-stage renal disease because of their higher baseline risk of bleeding due to thrombocytopathy-associated increased bleeding time, and possible bioaccumulation, despite research results to the contrary [36,52,53].

Interim Analyses
We will conduct 2 interim analyses, reviewed by an Independent Data Monitoring Committee who will examine blinded 2 group data. The primary outcome will be analyzed by the Haybitte-Peto Method using p=0.001 for each of the 2 interim analyses at one third and two thirds of projected total enrollment [54,55], controlling the overall type 1 error to 0.05, with the final analysis conducted at alpha=0.048. PROTECT has no provision for stopping early due to futility.

Participating Centers
We will randomize patients in several centers Canada, Australia, Brazil, Saudi Arabia, United States and United Kingdom.

Discussion

This manuscript has been written to transparently outline the implementation of the trial methods and the *a priori* analysis plans in advance of the results being available. The rigorous design suggests that the risk of systematic error is very low. The sample size suggests that the risk of random error is very low. PROTECT is the largest investigator-initiated, peer-review funded thromboprophylaxis trial in the world. It is the largest ICU trial funded by the Canadian Institutes for Health Research.

PROTECT results will have several important implications for clinical practice. If the results show that LMWH is more effective than UFH in medical-surgical critically ill patients, this trial will change practice in that LWMH may be the anticoagulant thromboprophylaxis of choice for this population. If the results show that UFH is as effective or more effective than LMWH, intensivists in many parts of the world may continue to use UFH while those currently using LMWH may reconsider and change to use UFH. Unfavourable consequences on major bleeding will also factor into such decisions.

We suggest that thromboprophylaxis decisions should be based on the best estimates of the absolute effects of different agents on the most patient-important thrombotic and bleeding outcomes, uncertainty around these estimates, and the availability and cost of the consequences of different agents in different jurisdictions.

Table 1

Intention to treat	No and Yes
Effectiveness (modified intention-to-treat, or as-treated)	No
Efficacy (per protocol)	No
Sensitivity analysis<br>(1) all post randomization events are considered incident<br>(2) only clinically suspected events are considered)	No
Subgroup analyses<br>(1) medical vs surgical patients<br>(2) patients receiving or not receiving vasopressors<br>(3) patients with end-stage dialysis dependence or not	No

Legend for Table 1
In this table we outline the structure to the basic analysis for PROTECT. All patients will be analyzed in the groups to which they are allocated. The intention-to-treat analysis will include all randomized patients except those from whom consent is withdrawn The effectiveness analysis (modified intention-to-treat, or as-treated) will include a smaller number of patients because patients will be excluded without bias for 3 reasons (please see text). The efficacy analysis (per-protocol) will include all patients who have at least 2 days of study drug, at least 2 technically adequate, legitimate VTE tests, and who do not have a prevalent VTE that is treated (please see text).

Acknowledgements
The trial was designed by the PROTECT Steering Committee, PROTECT Investigators and the Canadian Critical Care Trials Group. PROTECT was supported by the Canadian Critical Care Trials Group and the Australian and New Zealand Intensive Care Society Clinical Trials Group. Research Coordinators collected data. The Trial Biostatistician conducted the analyses. The Principal Investigator is the guarantor of this work.

This trial was funded by the Canadian Institute Health Research, Heart & Stroke Foundation of Canada and the Australian and New Zealand College of Anaesthetists Research Foundation. Pfizer Canada provided dalteparin; Eisai, Inc provided dalteparin for centers in the United States. None of these groups played a role in the design, conduct, analysis, interpretation or write-up of this trial.

D Cook holds a Chair of the Canadian Institutes of Health Research. M Meade is a Mentor of the Canadian Institutes of Health Research. TE Warkentin is supported by the Heart and Stroke Foundation of Canada. DJ Cooper is a National Health Medical Research Council Practitioner Fellow. M Crowther holds a Career Investigator Award from the Heart and Stroke Foundation of Ontario and the Leo Pharma Chair in Thromboembolism Research at McMaster University.

References

1 Attia J, Ray JG, Cook DJ, Douketis J, Ginsberg JS, Geerts W. Deep vein thrombosis and its prevention in critically ill patients. Arch Intern Med 2001;161(10):1268-1279.

2 Crowther MA, Cook DJ, Griffith LE et al. Deep venous thrombosis: clinically silent in the intensive care unit. J Crit Care 2005; 20(4):334-340.

3 Crowther MA, Cook DJ, Griffith LE et al. Neither baseline tests of molecular hypercoagulability nor D-dimer levels predict deep venous thrombosis in critically ill medical-surgical patients. Intensive Care Med 2005; 31(1):48-55.

4 Marik PE, Andrews L, Maini B. The incidence of deep venous thrombosis in ICU patients. Chest 1997;111:661-664.

5 Cook DJ, Crowther M, Meade M et al. Deep venous thrombosis in medical-surgical critically ill patients: Prevalence, incidence and risk factors. Crit Care Med 2005; 33(7):1565-1571.

6 McKelvie PA. Autopsy evidence of pulmonary thromboembolism. Med J Australia 1994;160:127-128.

7 Moser KM, LeMoine JR, Nachtwey FJ, Spragg RG. Deep venous thrombosis and pulmonary embolism: Frequency in a respiratory intensive care unit. JAMA 1981;246:1422-1424.

8 Hirsch DR, Ingenito EP, Goldhaber SZ. Prevalence of deep venous thrombosis among patients in medical intensive care. JAMA 1995;274:335-337.

9 Douketis JD, Kearon C, Bates S, Duku EK, Ginsberg JS. Risk of fatal pulmonary embolism in patients with treated venous thromboembolism. JAMA 1998;279:458-462.

10 Stein PD, Henry JW. Prevalence of acute pulmonary embolism among patients in a general hospital and at autopsy. Chest 1995;108;978-981.

11 Karwinski B, Svendsen E. Comparison of clinical and postmortem diagnosis of pulmonary embolism. J Clin Pathol 1989;42:135-139.

12 Twigg SJ, McCrirrick A, Sanderson PM. A comparison of post mortem findings with post hoc estimated clinical diagnoses of patients who die in a United Kingdom intensive care unit. Intensive Care Med 2001;27:706-710.

13 Clagett GP, Anderson FA, Geerts W, Heit J, Knudson MM, Lieberman JR, Merli GJ, Wheeler HB. Prevention of venous thromboembolism. Chest 1998;114:531S-560S.

14 Collins R, Scrimgeour A, Yusuf S et al. Reduction in fatal pulmonary embolism and venous thrombosis by perioperative administration of subcutaneous heparin. N Engl J Med 1988;318(18):1162-1173.

15 Geerts WH, Bergqviist D, Pineo GF, Heit JA, Samama CM, Lassen MR, Colwell C. Prevention of venous thromboembolism: American College of Chest Physicians Evidence Based Clinical Practice Guidelines (8th Edition). Chest 2008;133:381-453.

16 Cade JF. High risk of the critically ill for venous thromboembolism. Crit Care Med 1982;10:448-450.

17 Fraisse F, Holzapfel L, Couland JM, Simonneau G, Bedock B, Feissel H, Herbecq P, Pordes R, Poussel JF, Roux L. Nadroparin in the prevention of deep vein thrombosis in acute decompensated

COPD. Association of Non-University Affiliated Intensive Care Specialist Physicians in France. Am Rev Resp Crit Care Med 2000;161:1109-1114.

18 De A, Roy P, Garg VK et al. Low-molecular-weight heparin and unfractionated heparin in prophylaxis against deep vein thrombosis in critically ill patients undergoing major surgery. Blood Coagul Fibrinolysis 2010; 21(1):57-61.

19 Shorr AF, Williams MD. Venous thromboembolism in critically ill patients. Observations from a randomized trial in sepsis. Thromb Haemost 2009; 101(1):139-144.

20 Cruickshank MK, Levine MN, Hirsh J et al. An evaluation of impedance plethysmography and ^{125}I-fibrinogen leg scanning in patients following hip surgery. Thrombosis & Haemostasis1989;62:830-834.

21 Levi M, Levy M, William MD, Douglas I, Artigas A, Antonelli M et al for the Xigris in Prophylactic Heparin Evaluation in Severe Sepsis (XPRESS) Study Group. Prophylactic heparin in patients with severe sepsis treated with drotrecogin alfa (activated). Am Rev Resp Crit Care Med 2007;176:483-490,

22 Geerts WH, Jay RM, Code KI, Chen E, Szalai JP, Saibil EA, Hamilton PA. A comparison of low-dose heparin with low-molecular weight heparin as prophylaxis against venous thromboembolism after major trauma. N Engl J Med 1996;335:701-707.

23 Cook DJ, McMullin J, Hodder R, Heule M, Pinilla J, Dodek P, Stewart T, for the Canadian ICU Directors Group. Prevention and diagnosis of venous thromboembolism in critically ill patients: A Canadian survey. Crit Care 2001;5(6):336-342.

24 Cooper DJ, Bishop N, Cade J, Finfer S, Gallus A, Myburgh J for the ANZICS-CTG. Thromboprophylaxis in intensive care patients in Australia and New Zealand: A brief Survey Report. Crit Care 2005;20:354-356.

25 Cook DJ, Attia J, Weaver B, McDonald E, Meade M, Crowther M. Venous thromboembolic disease: An observational study in medical-surgical intensive care unit patients. J Crit Care 2000;15(4):127-132.

26 McMullin JP, Cook DJ, Griffith LE, McDonald E, Clarke F, Guyatt GH, Gibson J, Crowther M. Minimizing errors of omission: Behavioural rEenforcement of Heparin to Avert Venous Emboli (BEHAVE). Crit Care Med 2006; 34:694-699.

27 Robertson MS, Nichol AD, Higgins AM, Bailey MJ, Presneill JJ, Cooper DJ, Webb SA, McArthur C, MacIsaac CM, and the VTE Point Prevalence Investigators for the Australian and New Zealand Intensive Care Research Centre (ANZIC-RC) and the ANZICS-CTG. Venous thromboembolism prophylaxis in the critically ill: a point prevalence survey of current practice in Australian and New Zealand intensive care units. Critical Care and Resuscitation 2010;12(1):9-15

28 Lacherade JC, Cook DJ, Heyland D, Chrusch C, Brochard L, Brun-Buisson C, for the French and Canadian ICU Directors Groups. Prevention of venous thromboembolism in critically ill medical patients: A Franco-Canadian cross-sectional study. J Crit Care 2003;18(4):228-37.

29 Tapson VF, Decousus H, Pini M, Chong BH, Froehlich JB, Monreal M et al for the IMPROVE Investigators. Venous thromboembolism prophylaxis in acutely ill hospitalized medical patients: Findings from the international medical prevention registry on venous thromboembolism. Chest 207;132:936-945.

30 Cohen AT, Tapson V, Bergmann JF, Goldhaber SZ, Kakkar AK, Deslandes B et al for the ENDORSE Investigators. Venous thromboembolism risk and prophylaxis in the acute hospital setting (ENDORSE Study): a multinational cross-sectional study. Lancet 2008;371:387-394.

31 Goldhaber SZ. Venous thromboembolism in the intensive care unit: the last frontier for prophylaxis. [editorial]. Chest 1998;113:5-7.

32 Lederle FA. Heparin prophylaxis for medical patients? [editorial] Ann Intern Med 1998;128(9):768-770.

33 Evidence Report/Technology Assessment. Prevention of venous thromboembolism after injury. Agency for Health Care Policy Research (www.ahcpr.gov/clinic/vtsumm.htm)

34 Cook DJ, Rocker G, Meade MO, Guyatt GH, Geerts W, Anderson D et al for the PROTECT Investigators and the CCCTG. Prophylaxis of ThromboEmbolism in Critical Care Trials (PROTECT): A pilot study. J Crit Care 2005; 20:364-372.

35 Arnold DA, Burns KE, Adhikari N, Kho ME, Meade MO, Cook DJ. The design and interpretation of pilot trials in critical care medicine Crit Care Med 2009; 36(7):S69-S74

36 Douketis J, Cook DJ, Meade M, Guyatt G, Geerts W, Skrobik Y, Albert M, Granton J, Hebert PC, Pagliarello G, Marshall JC, Fowler R, Freitag A, Rabbat C, Anderson D, Zytaruk N, Heels-Ansdell D, Crowther MA for the Canadian Critical Care Trials Group. Renal insuffiency with the low-molecular-weight heparin dalteparin: An assessment of safety and pharmacodynamics. The DIRECT (Dalteparin's Influence on REnally Compromised anti-Ten-A) Study. Arch Intern Med 2008; 168(16):1805-1812.

37 Van Spaal HGC, Torren A, Kiss A, Fowler R. Eligibility criteria of randomized controlled trials published in high impact journals. JAMA 2007;297:1233-1244.

38 Warkentin TE, Greinacher A, Koster A, Lincoff AM. Treatment and prevention of heparin-induced thrombocytopenia. American College of Chest Physicians evidence-based clinical practice guidelines (8th edition). Chest 2008; 133 (6 Suppl.): 340S-380S

39 Sheridan D, Carter C, Kelton JG. A diagnostic test for heparin induced thrombocytopenia. Blood 1986;67:27-30.

40 Arnold DM, Donahoe L, Clarke FJ, Tkaczyk AJ, Heels-Ansdell D, Zytaruk N, Cook RJ, Webert KE, McDonald E, Cook DJ. Bleeding during critical illness: A prospective cohort study using a new measurement tool. Clin Invest Med 2007; 30:E93-E102.

41 Cook DJ, Medic A, Andrews J, Schiff D, Dobranowski J, Bates S, McDonald E, Landry F, Crowther AM. Doppler compression ultrasound of the lower extremities in ICU patients: An inter-rater reliability study. Crit Care Med 2003; A:346.

42 Southon J, Zytaruk N, Lim WP, Zarychanski R, Rocha M, D Heels-Ansdell, G Guyatt, Cook D, S Walter, for the PROTECT Investigators, CCCTG and ANZICS-CTG. Venous thrombosis adjudication for a thromboprophylaxis trial. Hamilton Research Day Abstract Book 2009

43 Lamontagne F, L McIntyre, P Dodek, J Southon, N Zytaruk, D Heels-Ansdell, M Meade, D Cook for the PROTECT Investigators, CCCTG and ANZICS-CTG. Calibration and adjudication of thrombotic events in a thromboprophylaxis trial. Canadian Critical Care Forum Abstract Book 2009.

44 Zytaruk N, Meade M, Mehta S, Hall R, Southon J, Heels-Ansdell D, Cook DJ for the PROTECT Investigators, CCCTG and ANZICS-CTG. Adjudication calibration for pulmonary embolism in a thromboprophylaxis trial. Am J Resp Crit Care Med 2009; 179:A1582

45 Arnold D, Rabbat C, Lauzier F, Zytaruk N, Heels-Ansdell D, Cook DJ, for the PROTECT Investigators and the CCCTG. Adjudicating bleeding outcomes in a large thromboprophylaxis trial in critical illness. Blood (ASH Annual Meeting Abstracts) 2009; 114: 2471.

46 Luce J, Cook DJ, Martin TR, Angus DC, Boushey HA, Curtis JR, Heffner JE, Lanen PN, Levy MM, Polite PY, Rocker GM, Truog RD for the Writing Committee of the American Thoracic Society Conference on the Ethical Conduct of Clinical Research Involving the Critically Ill. Luce J, Cook DJ, Martin T (Co-

chairs). The ethical conduct of clinical research involving critically ill patients in the United States and Canada: Principles and recommendations. Am J Resp Crit Care Med 2004; 170:1375-84.

47 Cook DJ, Lauzier F, Rocha MG, Sayles MJ, Finfer S. Serious adverse events in academic trials of common drugs in critical care Can Med Assoc J 2008; 178(9):1181-1184.

48 Baigent C, Harrel FH, Buyse M, Emberson JR, Altman DG. Ensuring trial validity by data quality assurance and diversification of monitoring methods. Sensible Guidelines Conference. Clinical Trials 2008;5:49-55.

49 Heels-Ansdell D, Walter S, Guyatt G, Warkentin T, Crowther Mark, Geerts W, Berwanger O, Rocha M, Freitag A, Cooper J, Qushmaq I, Zytaruk N, Cook DJ for the PROTECT Investigators, CCCTG and ANZICS-CTG. Central statistical monitoring in an international thromboprophylaxis trial. Am J Respir Crit Care Med 2010;181;A6041

50 DAMOCLES Study Group. A proposed charter for clinical trial data monitoring committees: helping them to do their job well. Lancet 2005;365:712-722.

51 Fergusson D, Aaron SD, Guyatt GH, Hébert P. Post-randomisation exclusions: the intention to treat principle and excluding patients from analysis BMJ 2002;325;652-654.

52 Nagge J, Crowther M, Hirsh J. Is impaired renal function a contraindication to the use of low molecular weight heparin? Arch Intern Med 2002;162:2605-2609.

53 Rabbat C, Cook DJ, Crowther MA, McDonald E, Clarke F, Meade MO, Lee KA, Cook RJ. Dalteparin thromboprophylaxis for critically ill medical-surgical patients. J Crit Care 2005; 20: 357-363.

54 Haybittle, JL. Repeated assessment of results in clinical trials of cancer treatment. British J Radiology 1971; 44: 793-7.

55 Peto R, Pike MC, Armitage P, Breslow NE, Cox DR, Howard SV, Mantel N, McPherson K, Peto J, Smith PG. Design and analysis of randomized clinical trials requiring prolonged observations of each patient. I. Introduction and design. British J Cancer 1976; 34: 585-612.
